# Therapeutic Effects of PADRE-BAFF Autovaccine on Rat Adjuvant Arthritis

**DOI:** 10.1155/2014/854954

**Published:** 2014-03-25

**Authors:** Guo-dong Feng, Xiao-chang Xue, Mei-li Gao, Xian-feng Wang, Zhen Shu, Nan Mu, Yuan Gao, Zeng-lu Wang, Qiang Hao, Wei-na Li, Meng Li, Cun Zhang, Wei Zhang, Ying-qi Zhang

**Affiliations:** ^1^State Key Laboratory of Cancer Biology, Department of Neurology, Xijing Hospital, Fourth Military Medical University, 17 Changle West Road, Shaanxi, Xi'an 710032, China; ^2^State Key Laboratory of Cancer Biology, Department of Biopharmaceutics, School of Pharmacy, Fourth Military Medical University, 17 Changle West Road, Xi'an 710032, China; ^3^The Biotechnology Center, Fourth Military Medical University, 17 Changle West Road, Xi'an, Shaanxi 710032, China; ^4^Key Laboratory of Biomedical Information Engineering of the Ministry of Education, Department of Biological Science and Engineering, School of Life Science and Technology, Xi'an Jiaotong University, Xi'an 710049, China; ^5^Department of Anesthesiology, Wake Forest University School of Medicine, Winston-Salem, NC 27157, USA

## Abstract

B cell activating factor (BAFF) is a cytokine of tumor necrosis factor family mainly produced by monocytes and dendritic cells. BAFF can regulate the proliferation, differentiation, and survival of B lymphocytes by binding with BAFF-R on B cell membrane. Accumulating evidences showed that BAFF played crucial roles and was overexpressed in various autoimmune diseases such as systemic lupus erythematous (SLE) and rheumatoid arthritis (RA). This suggests that BAFF may be a therapeutic target for these diseases. In the present study, we developed a BAFF therapeutic vaccine by coupling a T helper cell epitope AKFVAAWTLKAA (PADRE) to the N terminus of BAFF extracellular domains (PADRE-BAFF) and expressed this fusion protein in *Escherichia coli*. The purified vaccine can induce high titer of neutralizing BAFF antibodies and ameliorate the syndrome of complete Freund's adjuvant (CFA) induced rheumatoid arthritis in rats. Our data indicated that the BAFF autovaccine may be a useful candidate for the treatment of some autoimmune diseases associated with high level of BAFF.

## 1. Introduction

B cell activating factor (BAFF) is a member of tumor necrosis factor (TNF) family [1]. It is predominantly expressed by monocytes, macrophages, dendritic cells, and T cells [2, 3] and can form homotrimers or heterotrimers with a proliferation inducing ligand. BAFF is also responsible for regulating B cell maturation, survival, and function by binding to the receptors BAFF-R, TACI, or BCMA on B cells [4]. The active BAFF* in vivo* can occur in a soluble or membrane-bound form.

It has been shown that BAFF is a key regulator for B cell homeostasis [5]. B cell differentiation is severely perturbed in BAFF^−/−^ mice [6–8]; in contrast, BAFF transgenic mice develop autoimmune diseases resembling human SLE and Sjögren's syndrome [9–11]. Furthermore, overexpression of BAFF was found in sera of SLE and RA patients and BAFF/APRIL heterotrimers were also elevated in patients with various autoimmune conditions [12–15]. In light of the roles of BAFF in B cell function and these clinical data, BAFF may be regarded as a novel therapeutic target for the treatment of some human autoimmune diseases [16–18].

Rheumatoid arthritis (RA) is a chronic systemic inflammatory disorder that mainly affects the synovial joints but can also have systemic manifestations [19]. RA is characterized by hyperplasia of synovial cells, elevated cytokines and autoantibodies in synovial fluid, development of pannus in synovium, and infiltration of inflammatory lymphocytes including activated B cells [20]. Although the detailed mechanism of RA is still largely unknown, the progress in B cell-targeted therapies has greatly expanded our understanding of the critical role of B cells in RA pathogenesis [21, 22]. The contributions of B cells to antibody production, antigen presentation, T cell activation, and proinflammatory cytokines (such as TNF-*α*) secretion make BAFF a rational target for therapeutic reagents development in RA [17, 18].

In recent years, a novel vaccine construction strategy targeting self-proteins has been developed for treating various autoimmune diseases [23]. Immunological tolerance can be overcome by incorporating a T helper cell epitope within self-protein, which is helpful for eliciting therapeutic antibodies [24, 25]. PADRE T helper epitope (AKFVAAWTLKA) can bind to 15 of 16 of the most common HLA-DR types found to date with high or intermediate affinity. In this study, we constructed and expressed the therapeutic BAFF autovaccine by coupling the PADRE T helper epitope to the N terminus of BAFF (named PADRE-BAFF). Then, the therapeutic effect of the vaccine was investigated in the rat adjuvant arthritis (AA, an experiment animal model of RA). Our data demonstrated that high titer of antibodies was induced by the autovaccine in animals and that the induced antibodies could neutralize BAFF bioactivity both* in vitro* and* in vivo*, showing significant protective effects in AA animal model.

## 2. Materials and Methods

### 2.1. Reagents

Restriction endonucleases, Taq polymerase, and T4 DNA Ligase were purchased from TaKaRa (Dalian, China). Isopropyl-*β*-D-thiogalactoside (IPTG) was from Sigma (USA). Guanidine hydrochloride and Tris-HCl were from Serva (Germany). The pQE30 prokaryotic expression vector was stored in our laboratory. His-tag affinity column was obtained from Novagen (Germany). Five-liter fermentor was from B. Braun (Germany). Goat anti-human BAFF polyclonal antibody was from Santa Cruz Biotechnology (California, USA) and HRP-labeled rabbit anti-goat IgG was from Zhongshan Golden Bridge Biotechnology Co. Ltd. (Beijing, China). Complete and incomplete Freund's adjuvants were from Sigma (Saint Louis, Missouri, USA). Nucleotide oligos were synthesized by BioAsia (Shanghai, China). All other chemicals were of analytical grade.

### 2.2. Animals

Male Balb/c mice and Sprague-Dawley (SD) rats were purchased from the National Rodent Laboratory Animal Resource (Shanghai, China) and housed in a room with controlled ambient temperature (22 ± 2°C) and humidity (60 ± 5%). They were provided alternating 12 h periods of light and darkness. All procedures involving animals were approved by the Ethical Committee of the Institute and performed according to the Principles of Laboratory Animal Care (China).

### 2.3. Construction, Expression, and Purification of Recombinant PADRE-BAFF Molecules

The DNA coding for T helper epitope PADRE (AKFVAAWTLKA) was obtained by oligonucleotide synthesis according to the sequences 5′-GATCC ATGGCCAAATTTGTTGCTGCCTGGACCCTG AAAGCTG-3′ and 5′-AA T TCAGCTTTCAGGGTCCAGG CAGCAACAAATTTGGCCATG-3′. The sequences were designed to include* Bam*H I and* Eco*R I restriction sites (underlined). The gene of soluble BAFF was amplified from human leukocyte cDNA library with the following primers: 5′-GCGGAATTCGCCGTTC AGGGTCCAGAAG-3′ (forward) and 5′-GCGCTGCAGTCACAGCAGTTTC AATGCACCA-3′ (reverse).* Eco*R I and* Pst* I restriction sites are underlined. The PADRE oligonucleotides (annealed) and PCR products were inserted into pQE30 vector digested with* Bam*H I and* Pst* I. The construct of resulting plasmid pQPB (pQE30-PADRE-BAFF) was confirmed by DNA sequencing.

To express PADRE-BAFF protein, pQPB plasmid was transformed into BL21 (DE3) competent cells. A positive clone was inoculated into 5 mL Luria-Bertani (LB) medium supplemented with ampicillin (100 *μ*g/mL) and grown with 200 rpm shaking overnight at 37°C. Then, 50 *μ*L of culture was transferred into 5 mL of fresh LB medium and PADRE-BAFF expression was induced by adding 1 mM of IPTG when the OD_600 nm_ reached 0.5. After incubation at 37°C for 4 h, 1 mL of culture was collected and analyzed by SDS-PAGE to detect the expression of the recombinant vaccine.

For large scale purification, fermentation was performed using a 5 L fermentor as previously described [26]. The induced bacteria were harvested by centrifugation. Cell paste (100 g) was suspended in STE and sonicated on ice. Then, inclusion bodies (IBs) were solubilized in buffer A (7 M Guanidine HCl, 0.1 M NaH_2_PO_4_, and 0.01 M Tris-HCl pH 8.0) and loaded to a His-tag affinity chromatography column equilibrated with the same buffer. The column was eluted with buffer A and buffer B (7 M Guanidine HCl, 0.1 M NaH_2_PO_4_, and 0.01 M Tris-HCl pH 6.3) thoroughly to remove all unbound proteins and PADRE-BAFF was finally eluted with buffer B containing 250 mM imidazole. The purified IBs were refolded by dialysis against PBS at 20°C for 8 h twice. The refolded proteins were collected by centrifugation to remove the pellet and stored at −70°C for later use.

### 2.4. Characterization of Recombinant PADRE-BAFF

To analyze the purity of PADRE-BAFF, size exclusion chromatography- (SEC-) HPLC was performed on a Waters' HPLC system. The sample in PBS was injected into a 7.5 × 300 mm G2000SW column (TOSOH) at a flow rate of 0.5 mL/min and peaks were detected by monitoring at 280 nm. The purity of proteins was calculated as a percentage of the total peak area detected.

For Western blot, samples were transferred to PVDF membrane after SDS-PAGE using a wet transfer system. Western blot analysis was carried out using a goat anti-human BAFF polyclonal antibody, followed by an HRP-labeled rabbit anti-goat IgG. The target proteins were finally visualized with an enhanced chemiluminescence (ECL, Amersham).

The bioactivity of purified PADRE-BAFF was determined by assessing its effect on B cell line proliferation. In brief, Raji cells were collected and suspended at 2 × 10^6^/mL in RPMI 1640 medium supplemented with 10% heat-inactivated fetal calf serum (FCS). Cells were seeded in 96-well plates (2 × 10^5^/well) in triplicate and cultured in presence of 2 *μ*g/mL of goat F(ab′)_2_ anti-human IgM at 37°C in 5% CO_2_ in humid air. For proliferation assay, serially diluted PADRE-BAFF was added 6 h later, and standard BAFF and 1640 medium were used as positive and negative controls, respectively. After 66 h incubation, 20 *μ*L of MTT (0.5 mg/mL) was added and the plates were centrifuged (2000 rpm × 10 min⁡) 4 h later. The supernatants were discarded, and 100 *μ*L of DMSO was added. Samples were incubated at room temperature for 10 min and measured at 570 nm using a Bio-Rad plate reader to calculate the growth rate. Growth rate (%) = (OD  sample/OD  control) × 100%.

### 2.5. Induction of AA in Rats

The AA animal model was developed by inoculating 0.1 mL of complete Freund's adjuvant (CFA) into the hind metatarsal rat footpad of each foot. The day of CFA injection was designated day 0. From day 7, rats were examined daily for the onset of AA by assessing three parameters: body weight, paw swelling, and clinical score. Body weight was measured every other day as an indicator of systemic inflammation. For determination of paw swelling, the thickness of each hind paw was monitored three times a week by using a caliper. The clinical score was calculated on the basis of the following scoring system: 0, normal; 1, slight swelling and/or erythema; 2, moderate erythema and/or swelling; 3, severe erythema and/or swelling; 4, complete erythema and swelling of toes or fingers and ankle or wrist and inability to bend the ankle or wrist.

### 2.6. Immunization of Animals with PADRE-BAFF

PADRE-BAFF was dissolved in 0.9% saline (800 *μ*g/mL). Balb/c mice or SD rats were allocated to three groups (*n* = 10) and subcutaneously (s.c.) immunized with 20 *μ*g (mice) or 80 *μ*g (rats) of PADRE-BAFF with or without CFA, and saline was used in control groups. All animals were immunized at two-week intervals for three times followed by a boost given intraperitoneally (i.p.) at the sixth week. Sera were collected 10 days after the last immunization and analyzed by ELISA. Briefly, 96-well plates were coated with BAFF standards (100 ng/well) and incubated overnight at 4°C. Sera were diluted in a tenfold series (1 : 100–1 : 100,000) and loaded as primary antibodies. Peroxidase-conjugated goat anti-rat IgG was used as secondary antibody. All data were referred to a nonimmune serum and sera titers were assigned as the highest dilution that yielded an optical density greater than twice that of the nonimmune serum at the same dilution.

AA was induced when rats developed a BAFF-specific antibodies titer as high as 1 : 100,000 after the last immunization.

The neutralization assay was performed as previously described [27]. In brief, sera samples preincubated with standard BAFF at 37°C for 30 min were used in the Raji cell proliferation assay to investigate the blockade of BAFF effect by sera.

### 2.7. Statistical Analysis

All data were analyzed by the Student's *t*-tests, and differences were considered statistically significant when the *P* value was less than 0.05.

## 3. Results

### 3.1. Construction and Expression of PADRE-BAFF

**Figure 1:**
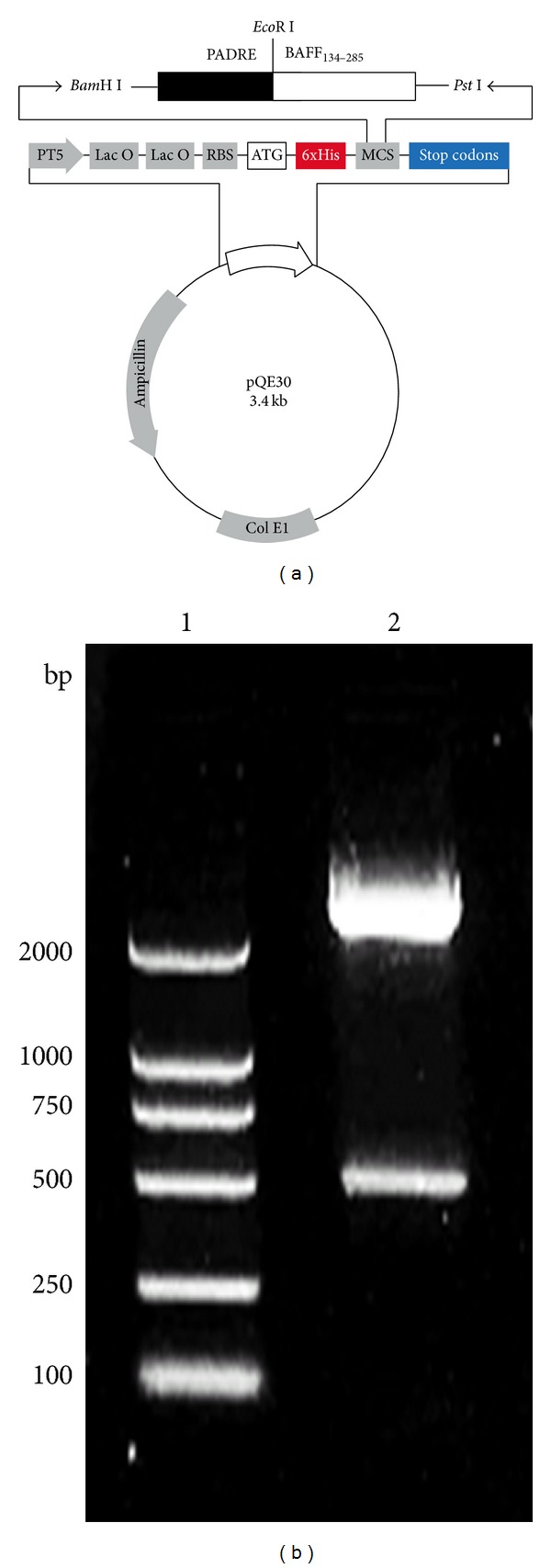
Schematic diagrams of pQE30-PADRE-BAFF expression plasmids. (a) The genes encoding PADRE-BAFF were cloned into pQE30 vector and expressed in* E. coli* BL21 under the control of T5 promoter and lac operator elements. (b) Analysis of recombinant plasmid pQE30-PADRE-BAFF by restriction enzyme digestion of Lane 1, DL2000 DNA marker; lane 2, pQE30-PADRE-BAFF digested with* Bam*H I and* Pst* I.

**Figure 2:**
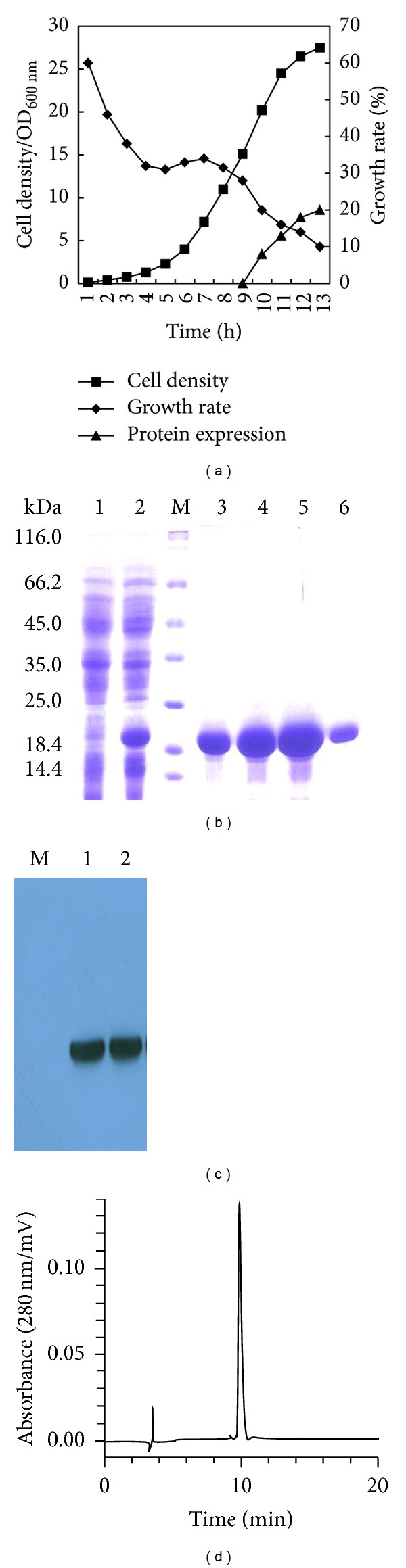
Production and identification of PADRE-BAFF. (a) Growth curve of engineered bacteria pQE30-PADRE-BAFF/BL21 (DE3) in fed-batch fermentation. (b) Expression and purification of PADRE-BAFF in* E. coli*. Lane 1, standard protein marker; lane 2, total bacterial proteins without induction; lane 3, total bacterial proteins with 1 mM IPTG induction; lanes 3–6, purification of PADRE-BAFF by chromatography. (c) Analysis of PADRE-BAFF by Western blot. Lane M, protein marker; lane 1, total proteins of pQE30-PADRE-BAFF/BL21 cells without induction; lane 2, total proteins of IPTG induced pQE30-PADRE-BAFF/BL21 cells. (d) Analysis of PADRE-BAFF by SEC-HPLC.

The recombinant PADRE-BAFF was constructed by PCR and gene synthesis. The coding sequence of extracellular fragment of BAFF was amplified by PCR from human leukocyte cDNA library and fused to chemically synthesized PADRE encoding DNA sequence at N terminus (Figure 1(a)). After identifying the plasmids by enzyme digest (Figure 1(b)) and DNA sequencing, the engineered bacterium pQPB/BL21 (DE3) was cultured in a 5 L fermentor and about 100 g wet weight of cells was obtained from 1 L of culture (Figure 2(a)). The expression of PADRE-BAFF was identified by SDS-PAGE. As shown in Figure 2(b), a new band was induced in the total proteins of pQPB/BL21 (DE3) strain compared with that of the BL21 (DE3) itself. And Western blot analysis showed that the new proteins induced by IPTG could be specifically recognized by goat anti-human BAFF antibody (Figure 2(c)).

### 3.2. Purification and Characterization of PADRE-BAFF

Recombinant PADRE-BAFF was purified by using a His-tag affinity chromatography column as described in Section 2 (Figure 2(b)). Then, the purified protein was refolded by dialysis and stored at −70°C.

The amino acid sequencing analysis showed that the N terminal sequences of PADRE-BAFF were as follow: MRGSHHHHHHAKFVA (data not shown), which was in agreement with the theoretical sequences of PADRE-BAFF as we designed. The purity of final purified protein was analyzed by SEC-HPLC and it is over 95% (Figure 2(d)).

### 3.3. Detection of Antibody Induced by PADRE-BAFF

**Figure 3:**
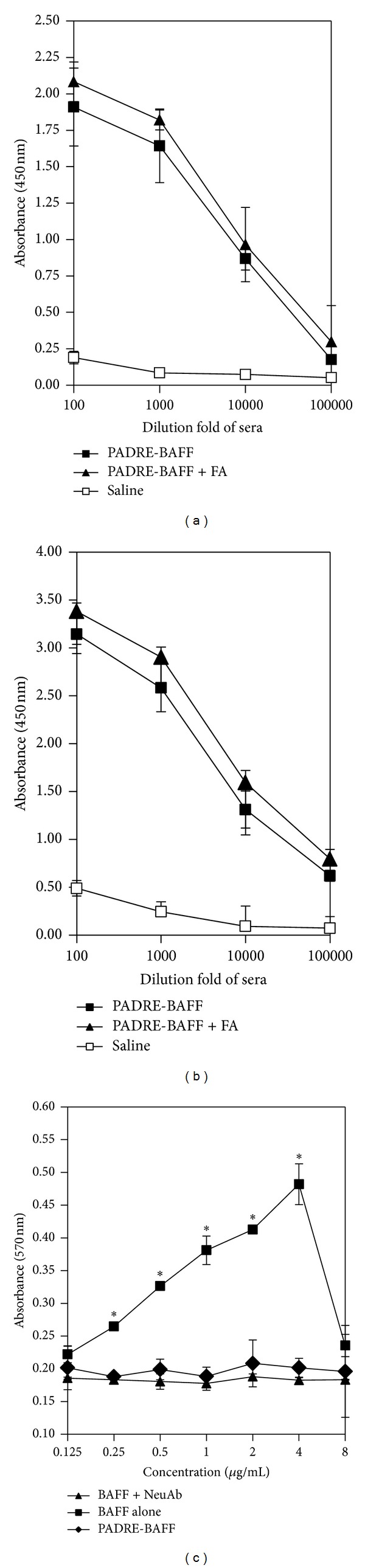
Serum antibody response of mice and rats immunized with PADRE-BAFF and neutralization assay. ((a), (b)) BAFF-specific serum antibody responses of mice (a) and rats (b) were measured by ELISA. Data represent the averages of triplicates obtained using sera after the last (fourth) administration of PADRE-BAFF. (c) Neutralizing antibody induced by PADRE-BAFF can block standard BAFF-stimulated Raji cell proliferation. *P < 0.01 compared with BAFF + NeuAb and PADRE-BAFF groups.

To determine whether the recombinant vaccine can effectively stimulate systemic immunity, Balb/c mice and SD rats were immunized s.c. at 2-week intervals with PADRE-BAFF. Sera were collected and specific serum binding antibodies against BAFF were detected by ELISA (Figures 3(a) and 3(b)). PADRE-BAFF could induce specific antibodies against BAFF and the antibody titer could reach a level of 1 : 100,000. Because of the high immunogenicity of PADRE, adjuvant has little effect on the immune response and no significant difference of the antibody titer can be seen between groups with or without Freund's adjuvant (*P* > 0.05). In order to investigate the neutralizing activity of induced polyclonal antibodies, antisera were heat-inactivated at 56°C for 30 min and then incubated with standard BAFF. Results showed that the proliferation of Raji cells stimulated by standard BAFF was apparently blocked by antisera (Figure 3(c)).

### 3.4. Therapeutic Effect of PADRE-BAFF on AA Animal Model

The polyclonal antibodies induced by PADRE-BAFF can block the bioactivity of standard BAFF* in vitro*; we go to determine whether the vaccine can effectively block endogenous BAFF* in vivo* and can be a therapeutic agent for BAFF overexpression associated autoimmune diseases. To this end, AA was induced in SD rats and the effect of PADRE-BAFF on this model was evaluated.

**Figure 4:**
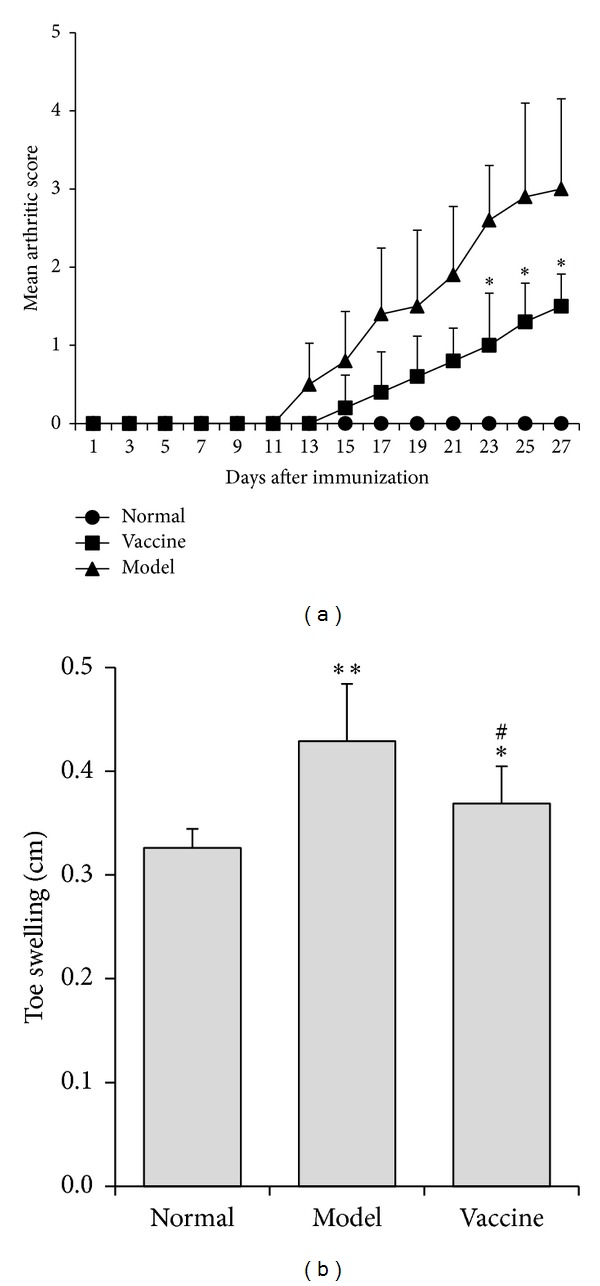
The clinical score of AA model in SD rats. (a) Thirty rats were subcutaneously immunized with PADRE-BAFF at two-week intervals for three times followed by a boost given intraperitoneally (i.p.). AA model was induced when the antibody titer was over 1 × 10^5^ and scored for clinical signs of RA as described in Section 2. Results are plotted as the mean clinical score ± SEM. The graph shows pooled data from three different experiments (10 rats per group). *P < 0.05 compared with normal group. (b) SD rats were immunized with PADRE-BAFF or saline and then AA model was induced and the paw swelling was measured with a caliper. Data are representative of three experiments. *P < 0.05, **P < 0.01, and ^#^ *P* < 0.05 compared with normal group.

SD rats were immunized four times with adjuvant-free PADRE-BAFF. One week after the last immunization, AA was induced as above. From day 7, rats were examined daily for the onset of AA by assessing body weight, paw swelling, and clinical score. As shown in Figure 4(a), arthritis symptoms can be obviously observed in both control and vaccine group rats, but the clinical symptoms of PADRE-BAFF-vaccinated mice were apparently ameliorated, as shown by the significant differences in the clinical score values.

**Figure 5:**
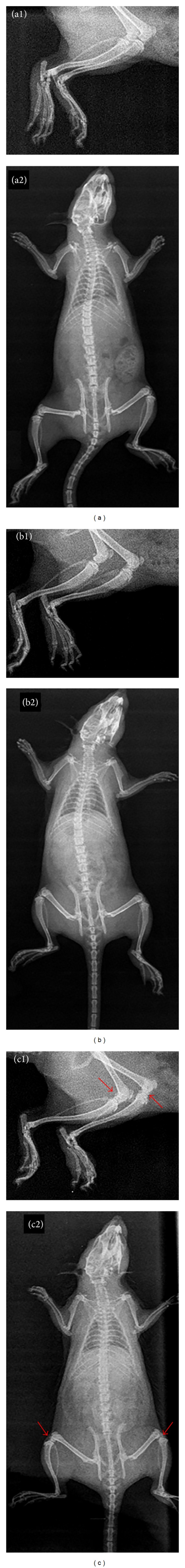
Representative X-ray images of rats. SD rats of normal (a), vaccine (b), and model (c) groups were anaesthetized and observed under X-ray at both left lateral position (top row) and frontal position (bottom row). Erosion in the articular cartilage was shown (arrow). All the rats were observed and these images are representative of all data.

In addition, the analysis of hind-paw swelling and the number of limbs involved indicated the amelioration of clinical symptoms in the PADRE-BAFF-vaccinated mice in comparison with the control mice (Figure 4(b)). We also examined the histologic changes in the knee joint by X-ray and images demonstrated an intense degree of decalcification and early erosion in the model group, which was not evident in the PADRE-BAFF-treated animals (Figure 5).

## 4. Discussion

It is well known that B cells play important roles in RA. B cell depletion therapy and dampened B cell subpopulations have shown beneficial effects in RA. As a result, B cell-related activation and survival genes have been paid more attention. BAFF is a potent regulator of B cell differentiation and survival [2, 5]. It is observed that B cell differentiation is severely perturbed in BAFF^−/−^ mice [6–8]. A recent study showed that BAFF gene expression and serum levels were the highest in the very early of untreated RA [28]. As more and more compelling direct evidence for BAFF overexpression in the sera as well as synovial fluid in RA patients has been gathered, BAFF is considered to be a candidate therapeutic target in RA treatment [14, 29, 30]. In this study, we constructed and expressed a BAFF autovaccine by coupling the extracellular domain of BAFF to the PADRE T helper cell epitope. We hope that the vaccine can instruct the exaggerated and uncontrolled humoral immune responses to produce BAFF neutralizing autoantibodies. And finally a new equilibrium will be reached and the immune system homeostasis will be rebuilt when the redundant endogenous BAFF and B cell subpopulations were removed.

Adjuvants are usually essential in autovaccines development, because it is usually difficult to effectively break tolerance against autopathological molecules and induce high titer of autoantibodies without an adjuvant. Unfortunately, adjuvants may have side effects in clinics [31]. For instance, the widely used aluminum adjuvants can cause pain, erythema, and swelling at the site of injection for several days [32]. Patients who receive aluminum adjuvants may show symptoms of myalgia, arthralgia, fatigue, or even serious neurological diseases such as multiple sclerosis [33]. Consequently, autovaccine immunization without adjuvants is more preferable. The autovaccine PADRE-BAFF that we used in this study owned high immunogenicity and can induce high titer of BAFF antibodies in mice and rats without adjuvant. When used in AA model in rats, adjuvant-free PADRE-BAFF apparently ameliorated the syndrome and had an obvious therapeutic action. Although we have evaluated the immunogenicity and preventive effects of PADRE-BAFF in animal models, before being used in clinical trials, the immunogenicity and therapeutic effects of this recombinant autovaccine should be systemically judged in different human tissue associated models. So we will further investigate its therapeutic effects in RA synovium/SCID chimeric model.

## 5. Conclusions

In the present study, we developed an adjuvant-free autovaccine of BAFF, a key molecule in regulating B lymphocyte homeostasis. The recombinant vaccine PADRE-BAFF was expressed in* E. coli* at high level as inclusion bodies. After purification and renaturation, the vaccine was used to immunize mice and rats and it can induce high titer of polyclonal antibodies to BAFF. We further evaluated the histologic changes, paw swelling, and clinical score of AA rats with or without PADRE-BAFF treatment; results showed that the adjuvant-free administration of the PADRE-BAFF vaccine apparently ameliorated symptoms of AA in rats. All these data demonstrate that the BAFF vaccines are useful for further research, especially for the* in vitro* preclinical evaluation of their potential as biological BAFF neutralizing agents.
